# Current Research on Non-Coding Ribonucleic Acid (RNA)

**DOI:** 10.3390/genes8120366

**Published:** 2017-12-05

**Authors:** Jing Wang, David C. Samuels, Shilin Zhao, Yu Xiang, Ying-Yong Zhao, Yan Guo

**Affiliations:** 1 Department of Biostatistics, Vanderbilt University, Medical Center, Nashville, TN 37232, USA; jing.wang.1@vanderbilt.edu (J.W.); Shilin.zhao.1@vanderbilt.edu (S.Z.); 2 Department of Molecular Physiology and Biophysics, Vanderbilt Genetics Institute, Vanderbilt University Medical School, Nashville, TN 37232, USA; david.c.samuels@vanderbilt.edu; 3 Department of Biochemistry and Molecular Biology, McGovern Medical School at The University of Texas Health Science Center at Houston, Houston, TX 77030, USA; Yu.Xiang@uth.tmc.edu; 4 Key Laboratory of Resource Biology and Biotechnology in Western China, School of Life Sciences, Northwest University, Xi’an 710069, Shaanxi, China; zyy@nwu.edu.cn; 5 Department of Internal Medicine, University of New Mexico, Albuquerque, NM 87102, USA

**Keywords:** long non-coding RNA, small RNA, detection, functional mechanism, disease, resource

## Abstract

Non-coding ribonucleic acid (RNA) has without a doubt captured the interest of biomedical researchers. The ability to screen the entire human genome with high-throughput sequencing technology has greatly enhanced the identification, annotation and prediction of the functionality of non-coding RNAs. In this review, we discuss the current landscape of non-coding RNA research and quantitative analysis. Non-coding RNA will be categorized into two major groups by size: long non-coding RNAs and small RNAs. In long non-coding RNA, we discuss regular long non-coding RNA, pseudogenes and circular RNA. In small RNA, we discuss miRNA, transfer RNA, piwi-interacting RNA, small nucleolar RNA, small nuclear RNA, Y RNA, single recognition particle RNA, and 7SK RNA. We elaborate on the origin, detection method, and potential association with disease, putative functional mechanisms, and public resources for these non-coding RNAs. We aim to provide readers with a complete overview of non-coding RNAs and incite additional interest in non-coding RNA research.

## 1. Introduction

It is commonly believed that protein-coding messenger ribonucleic acids (mRNAs) are the primary controller of cells, carrying out the necessary functions for life. Our understanding of how a gene functions has been significantly challenged due to the advances in high-throughput sequencing (HTS) technology, which has provided researchers with an expanded view of the complexity of the human genome, allowing for the identification of diverse types of RNAs. Mounting evidence from various studies has shown that the non-coding portion of the genome plays a more significant role in human biology than previously thought. Depending on the version of the annotation, the protein-coding genome regions represent around 1–3% of the human genome. However, the rest of the genome plays an important role in controlling the expression of the coding deoxyribonucleic acid (DNA), and in the spatial organization of the genome. One of the most critical corroborations of this is the distribution of results of genome-wide association studies (GWAS). Based on the latest GWAS Catalog (June 2017), only 3.56% of disease-associated single-nucleotide polymorphisms (SNPs) reside in the protein-coding region and 96.44% lie in non-coding regions equally proportioned between the intergenic regions and intron regions [1,2].

The Encyclopedia of DNA Elements (ENCODE) [3] is a large consortium project aimed at mapping all functional elements in the human genome. The initial results of the ENCODE project suggest that as much as 80% of the genome is biologically active and functional. However, this conclusion has drawn many sharp criticisms. The arguments against ENCODE’s conclusion include the C-value paradox [4], which argues that transcription activity does not necessarily indicate biological functionality [5], and the current estimated fraction of the genome that is conserved through purifying selection is less than 10% [6]. A later study was able to narrow down the fraction of the conserved region to 8.2% [7]. The argument between ENCODE and the other researchers who reject ENCODE’s conclusion primarily lies in the definition of functionality. For example, the encode researchers consider all DNAs that are transcribed to RNA including non-coding RNA as functional. However, some researchers only consider that evolutionarily conserved regions are functional.

**Figure 1:**
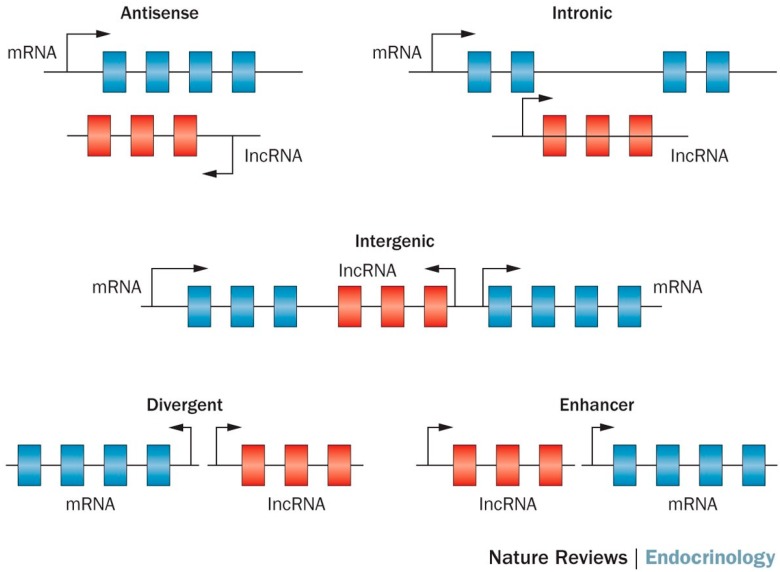
Biogenesis of long non-coding RNA (lncRNA) [10].

Non-coding RNA (ncRNA) is an RNA molecule that is transcribed from DNA but not translated to a protein. The last decade has witnessed a sharp rise of interest in non-coding RNA research (Figure 1). There are multiple classes and sub-classes of ncRNA. RNA size is a commonly used division of ncRNA: with small ncRNA (sRNA, less than 200 nucleotides), and long ncRNA (lncRNA, more than 200 nucleotides). Our review reflects on how the recent widespread interest in non-coding RNA is a result of the combination of the maturity of HTS technology and the bioinformatics development supporting that technology.

## 2. lncRNA

LncRNAs are a class of long transcribed but not translated RNAs that are longer than 200 nucleotides [8,9]. lncRNAs are often transcribed by RNA polymerase II, and can be classified as antisense, intronic, intergenic, divergent and enhancer lncRNAs according to their relative genome position (Figure 1) [10]. They have many characteristics similar to mRNAs such as 5’ capping, exon–intron splicing, and poly-adenylation, and the primary distinction from mRNAs is the lack of open reading frames (ORFs) [11]. LncRNA was discovered through the bioinformatics analysis of transcriptome data. Unlike protein coding mRNAs, lncRNA was traditionally believed to be non-functional. However, many recent studies have shown evidence for the functionality of lncRNA [9,12], such as roles in high-order chromosomal dynamics [13], embryonic stem cell differentiation [14], telomere biology [15] and subcellular structural organization [16]. The best characterized lncRNAs come from cancer studies. For example, MALAT-1 was associated with poor prognosis in non-small cell lung cancer [17,18], and oral squamous cell carcinoma [19]. Similarly, the lncRNA HOTAIR has been shown to promote metastasis in multiple cancers including breast, gastric, colorectal, cervical and liver cancer [20,21,22,23,24]. LncRNA SNHG1 has been found to regulate NOB1 expression by sponging miR-326 and promotes tumorigenesis in osteosarcoma [25]. Gioia R et al. observed that silencing lncRNA RP11-624C23.1 or RP11-203E8 could provide a selective advantage to leukemic cells by increasing resistance to genotoxic stress, possibly by modulating the DNA damage response (DDR) pathway [26]. Moreover, lncRNA was recently hailed as a possible biomarker for cancer [27,28,29]. For instance, lncRNA ZEB1-AS1 predicts unfavorable prognosis in gastric cancer [30] and lncRNA-ATB has potential as a biomarker for the prognosis of hepatocellular carcinoma and as a targeted therapy for afflicted patients [31].

**Figure 2:**
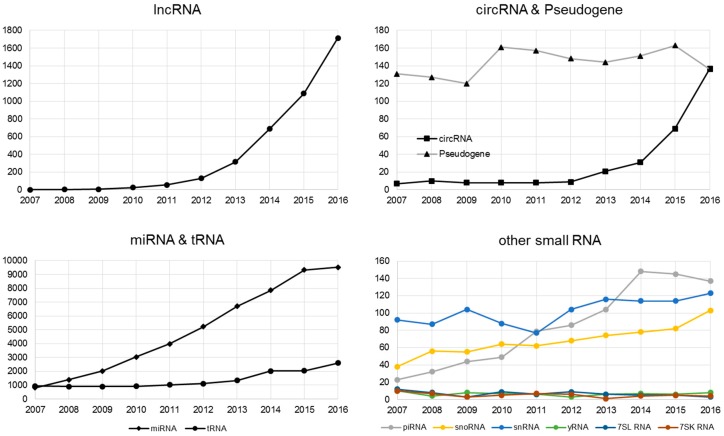
Sharp increase of publications focused on non-coding RNA by PubMed searches.

The interest in lncRNA has grown considerably as evidence of lncRNA’s role in various biological contexts has accumulated in recent years (Figure 2). lncRNAdb is a database that provides comprehensive annotations of eukaryotic lncRNAs manually curated from referenced literature [32]. The current version of lncRNAdb is released in Jan 2015 providing information of sequence, genomic context, expression profile, structure, subcellular localization, conservation, and function for 287 eukaryotic lncRNAs. LncRNADisease, another database that documents previously identified and predicts novel lncRNA-disease associations for human and mouse was published in 2013 [33]. The LncRNADisease curated the experimentally supported lncRNA-disease association data and integrated a tool for predicting potential associated diseases for a novel lncRNA based on its genomic context. In addition, LncRNADisease also collected lncRNA interactions in various levels, including protein, RNA, miRNA, and DNA. Current version (July 2017) of LncRNADisease documents ~3000 published lncRNA-disease associations including 914 lncRNAs and 329 diseases. Another notable lncRNA resource is LNCipedia (v4.1), a database which contains 146,742 annotated human lncRNAs [34]. Other published lncRNA database that are worth noting are lncRNome, a comprehensive database of lncRNA in humans [35]; MONOCLdb, which provides the annotations and expression profiles of mouse lncRNAs involved in influenza and SARS-CoV infections [36]; and NRED, a database of long non-coding RNA expression [37]. A list of currently available databases for lncRNA is shown in Table 1.

The identification of lncRNA requires the detection of transcription from unannotated genomic regions. This can be done by a number of techniques, including tiling array, serial analysis of gene expression (SAGE) [38], cap analysis gene expression (CAGE) [39], and the most powerful technique to date: RNA-seq, which prompts the development of multiple RNA-seq based pipelines for identifying lncRNAs [40,41]. Furthermore, chromatin immunoprecipitation (ChIP) technology, either ChIP-chip or Chip-seq can also identify novel lncRNAs indirectly by studying genomic regions with protein or histone modifications [42,43].

Traditional RNAseq libraries were built based on the presence of a poly(A) tail, which is present in all mRNA except for histone encoded mRNAs [44]. It is estimated that 60% of lncRNAs also have poly(A) tails [45]. Thus poly(A) tail-based RNA capture is not able to describe the entire range of lncRNAs. The preferred RNA library preparation method for this is total RNA library construction, which depletes ribosomal RNA (rRNA) and washes out small RNAs by size selection [46]. The current common total RNA library kits are Ribo-Zero and RNase H, each of which has its own strengths and weaknesses [47]. The total library usually requires more sequencing reads due to the multiple RNA species that are present in the library, and the rRNA reduction is not 100% efficient, usually leaving a portion of the rRNA in the library. In the last few years, lncRNA-focused microarray products have become available for purchase, such as Array star INC’s Human LncRNA Expression Microarray V4.0. However, these products are limited to known lncRNAs. In our opinion, RNAseq remains the ideal technology for detecting lncRNAs.

The number of definable lncRNAs varies by study. In 2005, a study claimed to have identified over 35,000 lncRNAs [11]. Another study in 2007 estimated that there are four times more lncRNA compared to protein-coding RNA [48]. In the ENCODE lncRNA release (Version 26), 15,787 lncRNAs were identified, further categorizing them into four sub-classes: antisense, large intergenic non-coding RNAs (lincRNA), sense intronic, and processed transcripts. In the latest study in 2017 by Hon et al., 27,919 human lncRNAs with high confidence 5’ end were described [49].

According to the latest gene annotation (GRCh38) gene transfer format file (GTF) (Homo_sapiens.GRCh38.89.gtf) from Ensemble, the total length of coding RNA is 97,662,789 nucleotides compared to 9,699,539 nucleotides for lincRNA. How lncRNA exerts its influence over disease is not well-known. The lncRNA-disease association may be activated through the regulation of protein-coding gene expression by a trans-expression quantitative trait locus (eQTL) and these trans-eQTL SNPs could be a surrogate SNP for SNPs residing in protein-coding RNA within the same high-linkage disequilibrium haplotype block. Cabili et al. found that many tissue-specific cis-eQTL are SNPs with known diseases or trait associations [50]. Hon et al. found that eQTL-linked lncRNA-mRNA pairs were more co-expressed than random lncRNA-mRNA pairs [49].

Even though the majority of lncRNAs are believed to be non-coding, some of them may potentially harbor novel unannotated proteins. In recent years, there have been increasing efforts to predict the functional potential of lncRNA. The first tool created for such a purpose was the Coding Potential Calculator (CPC) [51]. CPC assesses coding-potential by incorporating features such as the length of the open reading frame, coverage and integrity of the predicted open reading frame into a support vector machine (SVM). Getorf is a tool implemented by the European Molecular biology Open Software Suite [52], which identifies ORFs by identifying start and stop codons. The coding-potential assessment tool (CPAT) was designed to access coding potential through a logistic regression model accessing similar features used in CPC, such as length of ORF and ORF coverage. CPAT utilized two additional features, the Fickett score and the hexamer score, where the Fickett score describes the nucleotide composition of the RNA, and the hexamer score indicates the relative degree of hexamer usage bias in a particular sequence. The latest entry to this tool category is slncky [41], which is designed to discover lncRNAs from an RNA-seq dataset and assess their functional importance. Slncky assesses the coding potential by identifying conserved ORFs in syntenic regions across multiple species, with the syntenic regions identified by liftOver [53].

## 3. Circular RNA (circRNA)

**Figure 3:**
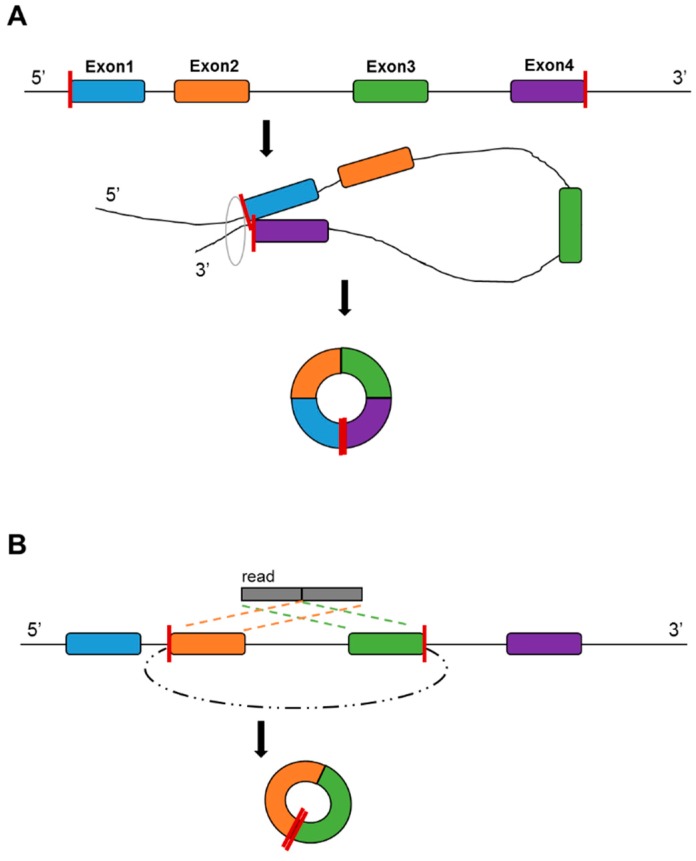
Formation of circular RNA. A. Direct ligation of 5’ and 3’ of the RNA; B. Backsplicing between exons.

CircRNA is a type of evolutionarily conserved RNA that forms a covalently closed continuous RNA loop. There are two scenarios of the formation of circRNAs: direct ligation of 5’ and 3’ ends of linear RNA, and backsplicing, wherein a downstream 3’ splice site joins to an upstream 5’ splice site (Figure 3). It has been suggested that the formation mechanism is associated with the RNA editing enzyme adenosine deaminase acting on RNA [54], and that the RNA-binding protein quaking regulates the formation of circRNAs [55]. It has been suggested that circRNA can be formed from mRNA and other non-coding RNA in the intergenic regions [56]. CircRNAs do not possess poly(A) tails [56,57], thus total RNA library preparation is more suitable for circRNA detection. There have been multiple reports that circRNA can be detected in non-tissue samples, such as saliva [58], blood plasma [59] and seminal plasma [60]. Currently, a major recognized function of circRNA is their action as a micro RNA (miRNA) sponge. It was found that circRNAs are much easier to bind with miRNA than with mRNA [61], allowing circRNA to repress miRNA regulation on mRNA.

CircRNA was originally identified through the analysis of scrambled exons over two decades ago using older, non-high throughput technology [62]. CircRNA was considered a rare event and exclusive to only a few viruses [63], and has evaded detection in mammalian genomes for a long time due to the lack of poly(A) tails. Advances in HTS technology have allowed researchers to scrutinize the mammalian genome in unprecedented detail, allowing for the identification of thousands of additional circRNAs. A large portion of the identified circRNAs are derived from protein-coding genes. However, after formation, these circRNAs are not able to undergo translation to proteins, and thus they have been categorized as non-coding RNA. Interestingly, recently several groups have discovered a protein-encoding function for some circRNAs, revealing an unexplored model of gene expression [64,65,66]. There have been increasingly more human disease studies devoted to circRNAs (Figure 2). For example, circRNA has been found to be a biomarker for cancer [67,68] and is associated with neurological disease [69]. The molecular roles and function, and how circRNA dysregulation affects disease, were reviewed in [70].

Novel bioinformatic tools have contributed greatly to the identification of the new circRNAs from RNA-seq data. Notable circRNA identification tools are find_circ [71], MapSplice2 [72], Segemehl [73], circExplorer [74], circRNA_finder [75], CIRI [76], ACFS [69], KNIFE [77], NCLscan [78], DCC [79] and UROBORUS [80]. Information regarding these tools is listed in Table 2. In addition, CircView, a platform visualization tool, is specifically developed to visualize circRNAs detected from these tools [81]. Furthermore, unlike other types of ncRNAs, circRNAs are not well-annotated. The traditional RNA annotation database RefSeq and Ensembl do not contain circRNA information currently. Instead, seven independent circRNA databases are available (Table 3) [82,83,84,85,86,87,88].

## 4. Pseudogenes

Pseudogenes are a category of ncRNA that resembles mRNA, but they are not transcribed into proteins. Currently there are two major mechanisms for the formation of pseudogenes. The first one suggests that pseudogenes are the products of the process of genomic duplication [89]. Genes in the duplicated regions often retain the original functions of their parent protein-coding genes. It was shown that high mutation rates were observed near or on duplicated regions [90]. When mutations such as stop-gain and frameshifts disrupt the original function of the duplicated genes, pseudogenes are formed. The second mechanism describes how pseudogenes can be formed through retrotransposition, where the process of reverse transcription of mRNA re-integrates cDNA sequences into the genome, forming new pseudogenes [91]. The formation of pseudogenes provides vital clues on how genomic DNA has adapted to evolutionary pressure to ensure survival. Pseudogenes are often regarded as dead or disabled with respect to protein synthesis. However, evolutionary studies have found evidence that a small number of pseudogenes in the human lineage have regained their protein-coding function [92].

The detection of pseudogenes usually relies on the careful analysis of sequence alignment. A common approach is to determine the homologous sequences to protein coding genes using tools such as FASTA or BLAST. Several more complicated computational approaches have been developed over the years to detect pseudogenes [93,94,95]. However, their utilization has been low, probably partially due to the lack of interest in pseudogenes. Despite this lack of enthusiasm, several interesting studies have shown how pseudogenes can potentially affect human health. Most of the proposed pseudogene functions are facilitated through the homology of their sequences. An example is PTENP1 which is the pseudogene of PTEN, a well-characterized tumor suppressor [96]. PTENP1 regulates cellular levels of PTEN by both sense and antisense RNAs which act as decoys for PTEN targeting microRNAs and also exert tumor-suppressive activities [97]. It has been shown that a missense mutation in PTENP1 can eliminate a codon associated with methionine initiation, thus inhibiting the translation of regular PTEN protein [97]. Another example is a pseudogene acting as a miRNA sponge. Studies have shown that BRAFP1 [98] and PTENP1 [97] compete for miRNA binding with their mRNA counterparts. The majority of the pseudogenes have been characterized in RefSeq and Ensembl. They are usually part of the regular RNA-seq analysis. Additional pseudogene resources for non-human species can be found at pseudogene.org [99]. Pseudofam provides family based pseudogene resources [100].

## 5. Small RNA

sRNAs are non-coding RNAs with a length of less than 200 nucleotides. The discovery of sRNAs has substantially enriched our understanding of the diverse world of RNAs. There are many species of sRNA. Here we will discuss eight species that have been proven to be detectable from HTS data; miRNA, transfer RNA (tRNA), piwi-interacting RNA (piRNA), small nucleolar RNA (snoRNA), small nuclear RNA (snRNA), Y RNA (yRNA), single-recognition particle RNA (7SL RNA), and 7SK RNA.

**Figure 4:**
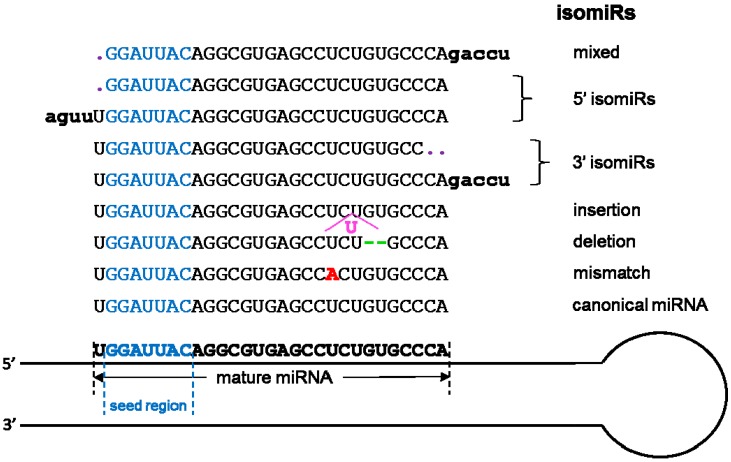
The most common scenarios for the formation of isomiRs. “Mixed” indicates that the formation of isomiRs can happen at both 3’ and 5’ ends.

The most studied smallest RNAs are miRNAs with more than 10,000 published manuscripts on PubMed (Figure 2). Originally discovered in 1993 [101], miRNAs are single-stranded ncRNAs of 19–25 nucleotides, which modulate translation by binding mRNAs through the seed sequence (up to seven nucleotides). Prior to the introduction of HTS technology, high-throughput miRNAs studies were conducted using hybridization-based technology, which limited the detection of miRNA to known and annotated miRNAs. The advancement of HTS has substantially increased the detection throughput of miRNA. More importantly, HTS enables the examination of miRNA at a single nucleotide resolution in addition to the quantification of abundance. By scrutinizing the precise nucleotide sequences of miRNA, researchers have discovered the phenomenon of miRNA isoforms (isomiR). The isomiRs are miRNAs with clipped seed sequences, compared to reference miRNA sequences [102] (Figure 4). The seed sequence of isomiRs and their parent miRNAs can differ by up to two nucleotides, causing a substantial difference in the repertoire of predicted mRNAs targets. Because miRNAs are well studied, their annotations are readily available in most annotation databases, such as Ensemble and Refseq. In addition, more than 50 miRNA and/or miRNA target resources are available now (Table 4 and Table 5). The most commonly used independent miRNA database is miRBase [103], and the most used miRNA target prediction webserver is TargetScan [104]. Another feature of miRNA that can be characterized by HTS technology is the non-templated nucleotide additions at the 3’ end of miRNAs [105,106,107,108]. The miRNA generally function as the transcriptional control of the regulatory elements of other protein-coding genes [109]. Thus, miRNAs play important roles in most biological processes, including development, proliferation, differentiation, immune reaction, apoptosis, tumorgenesis, adaptation to stress, and etc [101,109,110,111,112]. The miRNAs have exhibited potential as biomarkers or therapeutic targets for human diseases including cancer. For example, overexpression of miR-185 was shown to inhibit autophagy and apoptosis of dopaminergic cells in Parkinson’s disease, potentially via regulation of the AMPK/mTOR signaling pathway [113]. Several miRNAs have been repeatedly reported to be significantly dys-expressed in prostate cancer, including the down-regulated miR-143/145 and up-regulated let-7a, miR-130b, miR-141, and miR-17-5p [46,114,115]. In lung cancer, the 5q33 region containing miR-143 and miR-145 is often deleted which implies decreased expression of both miRNAs [116].

**Figure 5:**
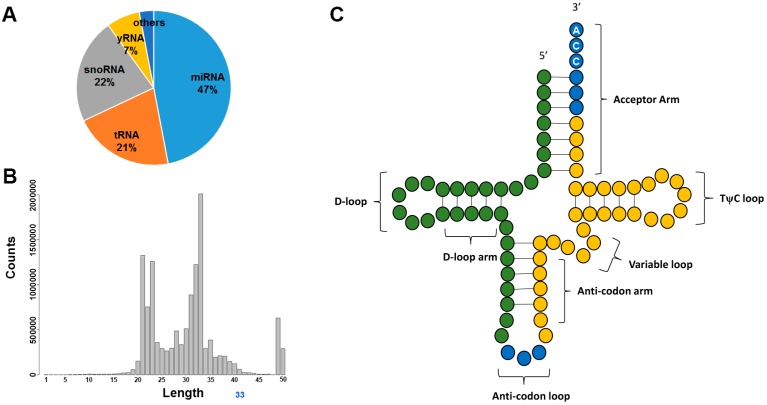
Small RNA (sRNA) sequencing. (**A**) The rough distribution of sRNA types in a sequencing sample. The distribution varies based on sample type; (**B**) example read length histogram after trimming adaptor sequences. Generally, two peaks (at 22 nucleotides for miRNA and at 33 nucleotides for transfer RNA (tRNA)) can be observed; (**C**) mature tRNA figure. The commonly captured fragments of tRNAs, forming the peaks in the length histogram, are the green and yellow segments of the tRNA.

HTS technology has also facilitated the discovery and identification of a wide range of sRNA species. At the infancy of HTS technology, small RNA-sequencing was often referred to as miRNA-sequencing as the goal was mostly to study miRNA. Through meticulous examination of HTS data, researchers became aware that miRNAs are only a fraction of the sRNA-sequencing data. The sequencing libraries for sRNA are constructed with size-selected gel electrophoresis, which is agnostic to sRNA categories. All RNA of less than 50 nucleotides in size are selected into the library. The most, or second most, abundant sRNA species is often tRNA (Figure 5A). tRNAs have a length of between 76 to 90 nucleotides, and serve as the physical link between mRNA and protein. The tRNAs detected with sRNA-sequencing are usually tRNA-derived sRNAs, a fragment of the parent tRNA, usually the 33 nucleotide sequences before the anticodon or the 33 nucleotide sequence after the anticodon (Figure 5B,C). The fragmentation of tRNA is caused by the cleavage by RNAse III enzyme, producing tRNA-derived halves [117,118]. Moreover, tRNAs can also be cleaved in a Dicer-dependent manner or as an in-vitro phenomenon by incubation with MgCl_2_ or nuclease S1 [119]. The 5’ tRNA fragments has been found to inhibit the translation initiation by interfering with the cap binding complex elF4F [120,121]. The production of the tRNA fragments has been shown to be associated with stress [118,122]. The detection of tRNA nucleotide variants and their association with diseases has also been increasingly reported [123,124,125,126]. For example, in animals, the tRNA fragment abundance has been found to be correlated with the severity of tissue damage in kidneys [127]. These 5’ tRNA fragments may be captured by high-throughput sequencing. In 2015, a novel technique was developed to sequence the entire tRNA by removing the bases with potential modification [128].

Other noticeable species of sRNA that can be detected through HTS are piRNA, yRNA, snRNA, snoRNA, 7SL, and 7SK. piRNA is a small RNA with a length of 24 to 32 nucleotides, and is considered by many to be the most abundant species of sRNA [129]. PiRNAs form RNA-protein complexes with the piwi proteins. Although the piRNA pathway has been commonly perceived as germline-specific, recent studies have demonstrated that the piRNA pathway has somatic functions [130], and potential associations with cancer [131]. Several studies have suggested that piRNA can be derived from pseudogenes [132,133]. yRNAs are components of the Ro60 ribonucleoprotein particle. Their primary function is DNA replication through interaction with chromatin and initiation proteins [134]. The latest studies have suggested that yRNAs regulate cell death and inflammation in monocytes [135]. snRNA is a species of small RNA confined to the splicing speckles and Cajal bodies of the nucleus in eukaryotic cells. The average length of snRNA is 150 nucleotides. The snRNAs bind with proteins to form small nuclear ribonucleoprotein particles (snRNPs). Some snRNAs are engaged in the formation and function of spliceosomes where pre-mRNA splicing occurs [136]. The transcription of snRNA is carried out by RNA polymerase II or III [137]. snoRNA is a species of sRNA with a length of 60 to 300 nucleotides. Its primary function is to guide chemical modifications of ribosomal RNA and tRNA. The two main classes of snoRNA are C/D box snoRNA and H/ACA box snoRNA. The primary function of C/D box snoRNA is to regulate methylation and pre-mRNA splicing [138]. The H/ACA box snoRNAs have been associated with pseudouridine, the most abundant modified nucleoside in RNA. Discovered in 1970 [139], 7SL is a species of sRNA, and a component of the signal-recognition particle ribonucleoprotein complex. The primary functions of 7SL RNA are to regulate protein translation [140], and post-translational transport [141]. 7SK is a species of sRNA found in metazoans. Its primary role is regulating transcription through the regulation of the positive transcription elongation factor P-TEFb [142].

Many miRNA processing pipelines have been established for utilizing HTS data. The major pipelines include: Oasis [143], Chimira [144], miRge [145], and TIGER [146], etc. Several tools have also been developed to detect isomiRs such as SeqBuster [147], isomiRID [148] and DeAnnIso [149]. One tRNA detection tool, tDRMapper [150], is currently available. For other species of sRNAs, the majority of the alignment-based pipelines would be sufficient for detection given that the annotation is available.

## 6. Conclusions

The biology of the human body is a vast and complex system and we are just beginning to understand the role of non-coding RNA in regulating that system. The advancement of biotechnology contributes to every breakthrough in our understanding of human biology. ncRNAs, the supposedly insignificant portion of the RNA universe, have exploded with an array of studies centered around the potential functions and disease associations related to ncRNAs. The sudden interest in ncRNA is due to the maturity of HTS technology and the development of bioinformatics allowing the interpretation of HTS data.

The interest in ncRNA is reflected by the increasing number of manuscripts published pertaining to ncRNA and the initiation of large consortium projects focused on ncRNA, such as ENCODE. The controversy surrounding whether ncRNAs are functional really comes down to the definition of “functional.” In the 2012 ENCODE publication [4], all transcribed RNAs were considered to be functional, but some researchers require a stricter definition. Nevertheless, the majority of the human genome should have a purpose, whether it is to synthesize proteins or to serve as a sponge for miRNA, or with lost or undiscovered mechanisms.

The common consensus of the functions of ncRNA is that they regulate gene expression at both transcriptional and epigenetic levels. The exact mechanism of this regulation varies by ncRNA categories; and some may be yet to be discovered. As HTS technology and sequencing library construction methods advance, we come closer to elucidating the entire human RNA spectrum and uncovering the secrets of ncRNAs.

## Figures and Tables

**Table 1:** lncRNA databases.

Name	Link	Dataset	Organism	PMID
ChIPBase v2.0	http://rna.sysu.edu.cn/chipbase/	transcriptional regulatory networks of ncRNAs and protein-coding genes	10 species	27924033
DIANA-LncBase v2	http://www.microrna.gr/LncBase/	miRNA-lncRNA interactions	human and mouse	26612864
LNCipedia 4.1	http://www.lncipedia.org/	annotated lncRNA transcripts	human	25378313
lncRNAdb v2.0	http://www.lncrnadb.org/	functional lncRNAs	68 species	25332394
LncRNADisease	http://www.cuilab.cn/lncrnadisease/	lncRNA-disease associations	human	23175614
LncRNAWiki	http://lncrna.big.ac.cn/	lncRNA knowledgebase	human	25399417
lncRNome	http://genome.igib.res.in/lncRNome/	lncRNA knowledgebase	human	23846593
MONOCLdb	http://www.monocldb.org/	lncRNAs expressed in collaborative cross founder mice in response to respiratory virus infection	mouse	24922324
NONCODE (v5.0)	http://www.noncode.org/	lncRNA knowledgebase	17 species	26586799
NRED	http://nred.matticklab.com/	lncRNA expression	human and mouse	18829717

**Table 2:** circRNA identification tools.

Tools	Language	Prerequisites	Input	PMID
find_circ	Python	Python (2.7) (numpy, pysam) Bowtie2 SAMtools	reads in fastq	23446348
MapSplice2	Python	g++ (≥4.3.3) Python (≥2.4.3) SAMtools Bowtie	reads in fasta/fastq	20802226
Segemehl	C	NA	reads in fasta	1975021224512684
circExplorer2	Python	Python (≥2.7) (pysam ≥ 0.8.4, pybedtools, pandas, docopt, and scipy) TopHat (≥2.0.9) Cufflinks (≥2.1.1) BEDTools genePredToGtf gtfToGenePred bedGraphToBigWig (optional) bedToBigBed (optional) STAR (≥2.4.0j) MapSplice (≥2.1.9) BWA (≥0.6.2-r126) segemehl (≥0.2.0)	reads in fastq	27365365
circRNA_Finder	Perl	Perl awk STAR (versions 2.4.1c) samtools	reads in fasta	25544350
CIRI	Perl	Perl	alignment in SAM	2558336528334140
ACFS	Perl	BWA-0.7.3a Perl BLAT (optional)	reads in fasta	27929140
KNIFE	Perl, Python & R	Bowtie2 (≥2.2.1) Bowtie (≥0.12.7) Perl Python 2.7.5 (numpy, scipy) R 3.0.2 (data.table) SAMtools	reads in fastq	26076956
NCLscan	Python & C++	Python 2.7 BEDTools SAMtools BLAT BWA	reads in fasta	26442529
DCC	Python	Python (pysam, pandas, numpy, and HTSeq)	alignment in BAM	26556385
UROBORUS	Perl	TopHat SAMtools Bowtie1/Bowtie2	alignment in SAM	26873924

**Table 3:** circRNA databases.

Name	Link	Dataset	Organism	PMID
circBase	http://www.circbase.org/	merged and unified data sets of circRNAs	Human, Mouse, Worm, fly, and coelacanth	25234927
circRNADb	http://202.195.183.4:8000/circrnadb/circRNADb.php	exonic circRNAs	Human	27725737
Circ2Traits	http://gyanxet-beta.com/circdb/	disease-circRNA association	Human	24339831
CircNet	http://circnet.mbc.nctu.edu.tw/	tissue-specific circRNA expression profiles and circRNA–miRNA-gene regulatory networks	Human	26450965
circRNABase (in starBase v2.0)	http://202.116.90.187/mirCircRNA.php	miRNA-circRNA interactions	Human, Mouse, and worm	24297251
PlantcircBase	http://ibi.zju.edu.cn/plantcircbase/	plant circular RNA Database	Rice, Mouse-ear cress, corn, Tomato, and Barley	28315753

**Table 4:** Webservers for microRNA (miRNA) target prediction.

Name	Link	Organism	PMID
ComiR	http://www.benoslab.pitt.edu/comir/	human, mouse, worm and fly	23703208
MBSTAR	http://www.isical.ac.in/~bioinfo_miu/MBStar30.htm	human	25614300
miRDB	http://www.mirdb.org/	human, mouse, rat, dog and chicken	25378301
miRmap	http://mirmap.ezlab.org/	human, mouse, cow, opossum, chicken, chimpanzee, zebrafish and other	23716633
mirPath v.3	http://www.microrna.gr/miRPathv3	human, mouse, rat, worm, fly, chicken and zebrafish	25977294
miRNA_Targets	http://mamsap.it.deakin.edu.au/mirna_targets/	human, mouse, chicken, cow, worm, fly and zebrafish	22940442
miRSystem	http://mirsystem.cgm.ntu.edu.tw/	human and mouse	22870325
miRTar2GO	http://www.mirtar2go.org/	human (cell type specific)	27903911
miRwalk2.0	http://zmf.umm.uni-heidelberg.de/mirwalk2	15 species	26226356
MR-microT	http://diana.imis.athena-innovation.gr/DianaTools/index.php?r=mrmicrot/index	human, mouse and fly	22285563
PITA	https://genie.weizmann.ac.il/pubs/mir07/mir07_prediction.html	human, mouse, fly and worm	17893677
psRNATarget	http://plantgrn.noble.org/psRNATarget/	plant	21622958
STarMir	http://sfold.wadsworth.org/starmir.html	mammalian	24803672
TAPIR	http://bioinformatics.psb.ugent.be/webtools/tapir	10 plant species	20430753
TargetScan v7.1	http://www.targetscan.org/vert_71/	human, mouse, worm, fly and fish	26267216
Tools4miRs	https://tools4mirs.org/	any	27153626

**Table 5:** miRNA databases.

Name	Link	Description	Organism	PMID
ChIPBase v2.0	http://rna.sysu.edu.cn/chipbase/	regulatory networks of ncRNAs and protein-coding genes	human	27924033
deepBase v2.0	http://biocenter.sysu.edu.cn/deepBase/	small RNAs, LncRNAs and circular RNAs	19 species	26590255
DIANA-TarBase v7.0	http://www.microrna.gr/tarbase	experimentally validated miRNA:gene interactions	24 species	25416803
doRiNA v2.0	http://dorina.mdc-berlin.de/	RNA interactions in post-transcriptional regulation	human, mouse, fly and worm	25416797
dPORE	http://cbrc.kaust.edu.sa/dpore	SNPs on the regulation of miRNAs and predicted TFBSs	human	21326606
Firefly Discovery Engine	https://www.fireflybio.com/portal/search	published miRNA papers	6 species	NA
HOCTAR2	http://hoctar.tigem.it/	miRNA target and expression	human	21435384
mESAdb	http://konulab.fen.bilkent.edu.tr/mirna/	sequences and expression of miRNA	human, mouse and zebrafish	21177657
microRNA.org	http://www.microrna.org	targets and expression	human, mouse, rat, fly and worm	18158296
miRBase	http://www.mirbase.org/	miRNA sequences and annotation	206 species	24275495
miRCancer	http://mircancer.ecu.edu/	miRNA Cancer Association	human	23325619
miRDB	http://www.mirdb.org/	targets and functional annotations	human, mouse, rat, dog and chicken	25378301
miRecords	http://miRecords.umn.edu/miRecords	animal miRNA-target interactions	9 species	18996891
miRGate	http://mirgate.bioinfo.cnio.es	miRNA-mRNA targets	human, mouse and rat	25858286
miRGator v3.0	http://mirgator.kobic.re.kr/	expression profiles and target relationships	human	23193297
miRGen v2.0	http://www.microrna.gr/mirgen	cell-line-specific miRNA TSSs and TF binding sites	human and mouse	26586797
miRmine	http://guanlab.ccmb.med.umich.edu/mirmine	miRNA expression profiles	human	28108447
miRNAMap v2.0	http://miRNAMap.mbc.nctu.edu.tw/	experimentally verified microRNAs and miRNA target genes	human, mouse, rat and other metazoan genomes	18029362
miRNEST v2.0	http://mirnest.amu.edu.pl	collection of animal, plant and virus microRNA data	544 species	24243848
miROrtho	http://cegg.unige.ch/mirortho	miRNA gene candidates	45 species	18927110
miRSel	http://services.bio.ifi.lmu.de/mirsel	literature derived miRNA-gene association	human, mouse and rat	20233441
miRTarBase v7.0	http://miRTarBase.mbc.nctu.edu.tw/	experimentally validated miRNA-target interactions	23 species	29126174
miRWalk v2.0	http://zmf.umm.uni-heidelberg.de/mirwalk2	predicted and experimentally verified miRNA-target interactions	15 species	26226356
PMRD	http://bioinformatics.cau.edu.cn/PMRD	plant microRNA	21 plant species	19808935
PolymiRTS Database	http://compbio.uthsc.edu/miRSNP/	DNA variations in miRNA seed regions and miRNA target sites	human and mouse	24163105
Psmir	http://www.bio-bigdata.com/Psmir/	potential associations between small molecules and miRNAs	human	26759061
RepTar	http://reptar.ekmd.huji.ac.il	predicted miRNA target	human and mouse	21149264
SonamiR DB v2.0	http://compbio.uthsc.edu/SomamiR/	cancer somatic mutations in miRNAs and their target sites	human	26578591
StarBase v2.0	http://starbase.sysu.edu.cn/	RNA-RNA and protein-RNA interaction networks from CLIP-Seq data	human, mouse and worm	24297251
STarMirDB	http://sfold.wadsworth.org/starmirDB.php	miRNA binding sites	human, mouse and worm	27144897
TargetScan v7.1	http://www.targetscan.org/vert_71/	miRNA targets	human, mouse, worm, fly and fish	26267216
^TM^REC	http://bioinfo.hrbmu.edu.cn/TMREC/	transcription factor and miRNA regulatory cascades in human diseases	human	25932650
TransmiR	http://cmbi.bjmu.edu.cn/transmir	transcription factor–microRNA regulation	14 species	19786497
VIRmiRNA	http://crdd.osdd.net/servers/virmirna	experimental viral miRNA and their targets	68 viruses	25380780

## References

[1] Freedman M.L., Monteiro A.N., Gayther S.A., Coetzee G.A., Risch A., Plass C., Casey G., De Biasi M., Carlson C., Duggan D. (2011). Principles for the post-GWAS functional characterization of cancer risk loci. Nat. Genet..

[2] Blattler A., Yao L., Witt H., Guo Y., Nicolet C.M., Berman B.P., Farnham P.J. (2014). Global loss of DNA methylation uncovers intronic enhancers in genes showing expression changes. Genome Biol..

[3] Consortium E.P., Bernstein B.E., Birney E., Dunham I., Green E.D., Gunter C., Snyder M. (2012). An integrated encyclopedia of DNA elements in the human genome. Nature.

[4] Eddy S.R. (2012). The C-value paradox, junk DNA and encode. Curr. Biol..

[5] Niu D.K., Jiang L. (2013). Can ENCODE tell us how much junk DNA we carry in our genome?. Biochem. Biophys. Res. Commun..

[6] Graur D., Zheng Y., Price N., Azevedo R.B., Zufall R.A., Elhaik E. (2013). On the immortality of television sets: “Function” in the human genome according to the evolution-free gospel of ENCODE. Genome Biol. Evol..

[7] Rands C.M., Meader S., Ponting C.P., Lunter G. (2014). 8.2% of the human genome is constrained: variation in rates of turnover across functional element classes in the human lineage. PLoS Genet..

[8] Perkel J.M. (2013). Visiting “noncodarnia”. Biotechniques.

[9] Mercer T.R., Dinger M.E., Mattick J.S. (2009). Long non-coding RNAs: insights into functions. Nat. Rev. Genet..

[10] Knoll M., Lodish H.F., Sun L. (2015). Long non-coding RNAs as regulators of the endocrine system. Nat. Rev. Endocrinol..

[11] Carninci P., Kasukawa T., Katayama S., Gough J., Frith M.C., Maeda N., Oyama R., Ravasi T., Lenhard B., Wells C. (2005). The transcriptional landscape of the mammalian genome. Science.

[12] Dinger M.E., Amaral P.P., Mercer T.R., Mattick J.S. (2009). Pervasive transcription of the eukaryotic genome: Functional indices and conceptual implications. Brief. Funct. Genom. Proteom..

[13] Amaral P.P., Mattick J.S. (2008). Noncoding RNA in development. Mamm. Genome.

[14] Dinger M.E., Amaral P.P., Mercer T.R., Pang K.C., Bruce S.J., Gardiner B.B., Askarian-Amiri M.E., Ru K., Solda G., Simons C. (2008). Long noncoding RNAs in mouse embryonic stem cell pluripotency and differentiation. Genome Res..

[15] Schoeftner S., Blasco M.A. (2008). Developmentally regulated transcription of mammalian telomeres by DNA-dependent RNA polymerase II. Nat. Cell Biol..

[16] Mercer T.R., Dinger M.E., Sunkin S.M., Mehler M.F., Mattick J.S. (2008). Specific expression of long noncoding RNAs in the mouse brain. Proc. Natl. Acad. Sci. USA.

[17] Schmidt L.H., Spieker T., Koschmieder S., Schaffers S., Humberg J., Jungen D., Bulk E., Hascher A., Wittmer D., Marra A. (2011). The long noncoding MALAT-1 RNA indicates a poor prognosis in non-small cell lung cancer and induces migration and tumor growth. J. Thorac. Oncol..

[18] Gutschner T., Hammerle M., Eissmann M., Hsu J., Kim Y., Hung G., Revenko A., Arun G., Stentrup M., Gross M. (2013). The noncoding RNA MALAT1 is a critical regulator of the metastasis phenotype of lung cancer cells. Cancer Res..

[19] Zhou X., Liu S., Cai G., Kong L., Zhang T., Ren Y., Wu Y., Mei M., Zhang L., Wang X. (2015). Long non coding RNA MALAT1 promotes tumor growth and metastasis by inducing epithelial-mesenchymal transition in oral squamous cell carcinoma. Sci. Rep..

[20] Gupta R.A., Shah N., Wang K.C., Kim J., Horlings H.M., Wong D.J., Tsai M.C., Hung T., Argani P., Rinn J.L. (2010). Long non-coding RNA HOTAIR reprograms chromatin state to promote cancer metastasis. Nature.

[21] Bhan A., Hussain I., Ansari K.I., Bobzean S.A., Perrotti L.I., Mandal S.S. (2014). Bisphenol-A and diethylstilbestrol exposure induces the expression of breast cancer associated long noncoding RNA HOTAIR in vitro and in vivo. J. Steroid Biochem. Mol. Biol..

[22] Bhan A., Hussain I., Ansari K.I., Kasiri S., Bashyal A., Mandal S.S. (2013). Antisense transcript long noncoding RNA (lncRNA) HOTAIR is transcriptionally induced by estradiol. J. Mol. Biol..

[23] Hajjari M., Salavaty A. (2015). Hotair: An oncogenic long non-coding RNA in different cancers. Cancer Biol. Med..

[24] Zhong D.N., Luo Y.H., Mo W.J., Zhang X., Tan Z., Zhao N., Pang S.M., Chen G., Rong M.H., Tang W. (2017). High expression of long noncoding HOTAIR correlated with hepatocarcinogenesis and metastasis. Mol. Med. Rep..

[25] Wang J., Cao L., Wu J., Wang Q. (2017). Long non-coding RNA SNHG1 regulates NOB1 expression by sponging miR-326 and promotes tumorigenesis in osteosarcoma. Int. J. Oncol..

[26] Gioia R., Drouin S., Ouimet M., Caron M., St-Onge P., Richer C., Sinnett D. (2017). LncRNAs downregulated in childhood acute lymphoblastic leukemia modulate apoptosis, cell migration, and DNA damage response. Oncotarget.

[27] Shi T., Gao G., Cao Y. (2016). Long noncoding RNAs as novel biomarkers have a promising future in cancer diagnostics. Dis. Markers.

[28] Yarmishyn A.A., Kurochkin I.V. (2015). Long noncoding RNAs: A potential novel class of cancer biomarkers. Front. Genet..

[29] Tian S., Rulli S., Lader E. (2015). Serum lncRNA detection as potential biomarker of lung cancer. FASEB J..

[30] Li Y., Wen X., Wang L., Sun X., Ma H., Fu Z., Li L. (2017). LncRNA ZEB1-AS1 predicts unfavorable prognosis in gastric cancer. Surg. Oncol..

[31] Jang S.Y., Kim G., Park S.Y., Lee Y.R., Kwon S.H., Kim H.S., Yoon J.S., Lee J.S., Kweon Y.-O., Ha H.T. (2017). Clinical significance of lncRNA-ATB expression in human hepatocellular carcinoma. Oncotarget.

[32] Quek X.C., Thomson D.W., Maag J.L., Bartonicek N., Signal B., Clark M.B., Gloss B.S., Dinger M.E. (2015). LncRNAdb v2.0: Expanding the reference database for functional long noncoding RNAs. Nucleic Acids Res..

[33] Chen G., Wang Z., Wang D., Qiu C., Liu M., Chen X., Zhang Q., Yan G., Cui Q. (2013). LncRNADisease: A database for long-non-coding RNA-associated diseases. Nucleic Acids Res..

[34] Volders P.J., Verheggen K., Menschaert G., Vandepoele K., Martens L., Vandesompele J., Mestdagh P. (2015). An update on LNCipedia: A database for annotated human lncRNA sequences. Nucleic Acids Res..

[35] Bhartiya D., Pal K., Ghosh S., Kapoor S., Jalali S., Panwar B., Jain S., Sati S., Sengupta S., Sachidanandan C. (2013). lncRNome: a comprehensive knowledgebase of human long noncoding RNAs. Database (Oxford).

[36] Josset L., Tchitchek N., Gralinski L.E., Ferris M.T., Eisfeld A.J., Green R.R., Thomas M.J., Tisoncik-Go J., Schroth G.P., Kawaoka Y. (2014). Annotation of long non-coding RNAs expressed in collaborative cross founder mice in response to respiratory virus infection reveals a new class of interferon-stimulated transcripts. RNA Biol..

[37] Dinger M.E., Pang K.C., Mercer T.R., Crowe M.L., Grimmond S.M., Mattick J.S. (2009). NRED: A database of long noncoding RNA expression. Nucleic Acids Res..

[38] Velculescu V.E., Zhang L., Vogelstein B., Kinzler K.W. (1995). Serial analysis of gene expression. Science.

[39] Shiraki T., Kondo S., Katayama S., Waki K., Kasukawa T., Kawaji H., Kodzius R., Watahiki A., Nakamura M., Arakawa T. (2003). Cap analysis gene expression for high-throughput analysis of transcriptional starting point and identification of promoter usage. Proc. Natl. Acad. Sci. USA.

[40] Sun L., Zhang Z., Bailey T.L., Perkins A.C., Tallack M.R., Xu Z., Liu H. (2012). Prediction of novel long non-coding RNAs based on RNA-Seq data of mouse Klf1 knockout study. BMC Bioinform..

[41] Chen J., Shishkin A.A., Zhu X.P., Kadri S., Maza I., Guttman M., Hanna J.H., Regev A., Garber M. (2016). Evolutionary analysis across mammals reveals distinct classes of long non-coding RNAs. Genome Biol..

[42] Guttman M., Amit I., Garber M., French C., Lin M.F., Feldser D., Huarte M., Zuk O., Carey B.W., Cassady J.P. (2009). Chromatin signature reveals over a thousand highly conserved large non-coding RNAs in mammals. Nature.

[43] Cottrell K.A., Djuranovic S. (2016). Urb-RIP - an adaptable and efficient approach for immunoprecipitation of RNAs and associated RNAs/proteins. PLoS ONE.

[44] Guo Y., Zhao S., Sheng Q., Guo M., Lehmann B., Pietenpol J., Samuels D.C., Shyr Y. (2015). RNAseq by total RNA library identifies additional RNAs compared to poly(a) RNA library. BioMed Res. Int..

[45] Cheng J., Kapranov P., Drenkow J., Dike S., Brubaker S., Patel S., Long J., Stern D., Tammana H., Helt G. (2005). Transcriptional maps of 10 human chromosomes at 5-nucleotide resolution. Science.

[46] Song C., Chen H., Wang T., Zhang W., Ru G., Lang J. (2015). Expression profile analysis of microRNAs in prostate cancer by next-generation sequencing. Prostate.

[47] Guo Y., Wu J., Zhao S., Ye F., Su Y., Clark T., Sheng Q., Lehmann B., Shu X.O., Cai Q. (2016). RNA sequencing of formalin-fixed, paraffin-embedded specimens for gene expression quantification and data mining. Int. J. Genom..

[48] Kapranov P., Cheng J., Dike S., Nix D.A., Duttagupta R., Willingham A.T., Stadler P.F., Hertel J., Hackermuller J., Hofacker I.L. (2007). RNA maps reveal new RNA classes and a possible function for pervasive transcription. Science.

[49] Hon C.C., Ramilowski J.A., Harshbarger J., Bertin N., Rackham O.J., Gough J., Denisenko E., Schmeier S., Poulsen T.M., Severin J. (2017). An atlas of human long non-coding RNAs with accurate 5’ ends. Nature.

[50] Cabili M.N., Trapnell C., Goff L., Koziol M., Tazon-Vega B., Regev A., Rinn J.L. (2011). Integrative annotation of human large intergenic noncoding RNAs reveals global properties and specific subclasses. Genes Dev..

[51] Kong L., Zhang Y., Ye Z.Q., Liu X.Q., Zhao S.Q., Wei L., Gao G. (2007). CPC: Assess the protein-coding potential of transcripts using sequence features and support vector machine. Nucleic Acids Res..

[52] Rice P., Longden I., Bleasby A. (2000). EMBOSS: The European Molecular Biology Open Software Suite. Trends Genet..

[53] Hinrichs A.S., Karolchik D., Baertsch R., Barber G.P., Bejerano G., Clawson H., Diekhans M., Furey T.S., Harte R.A., Hsu F. (2006). The UCSC genome browser database: update 2006. Nucleic Acids Res..

[54] Ivanov A., Memczak S., Wyler E., Torti F., Porath H.T., Orejuela M.R., Piechotta M., Levanon E.Y., Landthaler M., Dieterich C. (2015). Analysis of intron sequences reveals hallmarks of circular RNA biogenesis in animals. Cell Rep..

[55] Conn S.J., Pillman K.A., Toubia J., Conn V.M., Salmanidis M., Phillips C.A., Roslan S., Schreiber A.W., Gregory P.A., Goodall G.J. (2015). The RNA binding protein quaking regulates formation of circRNAs. Cell.

[56] Chen I., Chen C.Y., Chuang T.J. (2015). Biogenesis, identification, and function of exonic circular RNAs. Wiley Interdiscip. Rev. RNA.

[57] Jeck W.R., Sorrentino J.A., Wang K., Slevin M.K., Burd C.E., Liu J.Z., Marzluff W.F., Sharpless N.E. (2013). Circular RNAs are abundant, conserved, and associated with ALU repeats RNA 2013, 19, 426. RNA.

[58] Bahn J.H., Zhang Q., Li F., Chan T.M., Lin X., Kim Y., Wong D.T.W., Xiao X. (2015). The landscape of microRNA, Piwi-interacting RNA, and circular RNA in human saliva. Clin. Chem..

[59] Koh W., Pan W., Gawad C., Fan H.C., Kerchner G.A., Wyss-Coray T., Blumenfeld Y.J., El-Sayed Y.Y., Quake S.R. (2014). Noninvasive in vivo monitoring of tissue-specific global gene expression in humans. Proc. Natl. Acad. Sci. USA.

[60] Dong W.W., Li H.M., Qing X.R., Huang D.H., Li H.G. (2016). Identification and characterization of human testis derived circular RNAs and their existence in seminal plasma. Sci. Rep..

[61] Hansen T.B., Jensen T.I., Clausen B.H., Bramsen J.B., Finsen B., Damgaard C.K., Kjems J. (2013). Natural RNA circles function as efficient microRNA sponges. Nature.

[62] Nigro J.M., Cho K.R., Fearon E.R., Kern S.E., Ruppert J.M., Oliner J.D., Kinzler K.W., Vogelstein B. (1991). Scrambled exons. Cell.

[63] Jeck W.R., Sharpless N.E. (2014). Detecting and characterizing circular RNAs. Nat. Biotechnol..

[64] Yang Y., Fan X., Mao M., Song X., Wu P., Zhang Y., Jin Y., Yang Y., Chen L.L., Wang Y. (2017). Extensive translation of circular RNAs driven by N6-methyladenosine. Cell Res..

[65] Pamudurti N.R., Bartok O., Jens M., Ashwal-Fluss R., Stottmeister C., Ruhe L., Hanan M., Wyler E., Perez-Hernandez D., Ramberger E. (2017). Translation of circRNAs. Mol. Cell.

[66] Legnini I., Di Timoteo G., Rossi F., Morlando M., Briganti F., Sthandier O., Fatica A., Santini T., Andronache A., Wade M. (2017). Circ-ZNF609 is a circular RNA that can be translated and functions in myogenesis. Mol. Cell.

[67] Qin M.L., Liu G., Huo X.S., Tao X.M., Sun X.M., Ge Z.H., Yang J., Fan J., Liu L., Qin W.X. (2016). Hsa_circ_0001649: A circular RNA and potential novel biomarker for hepatocellular carcinoma. Cancer Biomark..

[68] Zhang Y., Liang W., Zhang P., Chen J., Qian H., Zhang X., Xu W. (2017). Circular RNAs: Emerging cancer biomarkers and targets. J. Exp. Clin. Cancer Res..

[69] You X.T., Vlatkovic I., Babic A., Will T., Epstein I., Tushev G., Akbalik G., Wang M.T., Glock C., Quedenau C. (2015). Neural circular RNAs are derived from synaptic genes and regulated by development and plasticity. Nat. Neurosci..

[70] Holdt L.M., Kohlmaier A., Teupser D. (2017). Molecular roles and function of circular RNAs in eukaryotic cells. Cell. Mol. Life Sci..

[71] Memczak S., Jens M., Elefsinioti A., Torti F., Krueger J., Rybak A., Maier L., Mackowiak S.D., Gregersen L.H., Munschauer M. (2013). Circular RNAs are a large class of animal RNAs with regulatory potency. Nature.

[72] Wang K., Singh D., Zeng Z., Coleman S.J., Huang Y., Savich G.L., He X., Mieczkowski P., Grimm S.A., Perou C.M. (2010). MapSplice: Accurate mapping of RNA-seq reads for splice junction discovery. Nucleic Acids Res..

[73] Hoffmann S., Otto C., Doose G., Tanzer A., Langenberger D., Christ S., Kunz M., Holdt L.M., Teupser D., Hackermuller J. (2014). A multi-split mapping algorithm for circular RNA, splicing, trans-splicing and fusion detection. Genome Biol..

[74] Zhang X.O., Wang H.B., Zhang Y., Lu X., Chen L.L., Yang L. (2014). Complementary sequence-mediated exon circularization. Cell.

[75] Westholm J.O., Miura P., Olson S., Shenker S., Joseph B., Sanfilippo P., Celniker S.E., Graveley B.R., Lai E.C. (2014). Genome-wide analysis of drosophila circular RNAs reveals their structural and sequence properties and age-dependent neural accumulation. Cell Rep..

[76] Gao Y., Wang J., Zhao F. (2015). CIRI: An efficient and unbiased algorithm for de novo circular RNA identification. Genome Biol..

[77] Szabo L., Morey R., Palpant N.J., Wang P.L., Afari N., Jiang C., Parast M.M., Murry C.E., Laurent L.C., Salzman J. (2015). Statistically based splicing detection reveals neural enrichment and tissue-specific induction of circular RNA during human fetal development. Genome Biol..

[78] Chuang T.J., Wu C.S., Chen C.Y., Hung L.Y., Chiang T.W., Yang M.Y. (2016). NCLscan: Accurate identification of non-co-linear transcripts (fusion, trans-splicing and circular RNA) with a good balance between sensitivity and precision. Nucleic Acids Res..

[79] Cheng J., Metge F., Dieterich C. (2016). Specific identification and quantification of circular RNAs from sequencing data. Bioinformatics.

[80] Song X., Zhang N., Han P., Moon B.S., Lai R.K., Wang K., Lu W. (2016). Circular RNA profile in gliomas revealed by identification tool UROBORUS. Nucleic Acids Res..

[81] Feng J., Xiang Y., Xia S., Liu H., Wang J., Ozguc F.M., Lei L., Kong R., Diao L., He C. (2017). CircView: A visualization and exploration tool for circular RNAs. Brief. Bioinform..

[82] Glazar P., Papavasileiou P., Rajewsky N. (2014). circBase: A database for circular RNAs. RNA.

[83] Chen X., Han P., Zhou T., Guo X., Song X., Li Y. (2016). CircRNADb: A comprehensive database for human circular RNAs with protein-coding annotations. Sci. Rep..

[84] Ghosal S., Das S., Sen R., Basak P., Chakrabarti J. (2013). Circ2Traits: A comprehensive database for circular RNA potentially associated with disease and traits. Front. Genet..

[85] Liu Y.C., Li J.R., Sun C.H., Andrews E., Chao R.F., Lin F.M., Weng S.L., Hsu S.D., Huang C.C., Cheng C. (2016). CircNet: A database of circular RNAs derived from transcriptome sequencing data. Nucleic Acids Res..

[86] Li J.H., Liu S., Zhou H., Qu L.H., Yang J.H. (2014). starBase v2.0: Decoding miRNA-ceRNA, miRNA-ncRNA and protein-RNA interaction networks from large-scale CLIP-Seq data. Nucleic Acids Res..

[87] Chu Q., Zhang X., Zhu X., Liu C., Mao L., Ye C., Zhu Q.H., Fan L. (2017). PlantcircBase: A database for plant circular RNAs. Mol. Plant.

[88] Xia S., Feng J., Lei L., Hu J., Xia L., Wang J., Xiang Y., Liu L., Zhong S., Han L. (2016). Comprehensive characterization of tissue-specific circular RNAs in the human and mouse genomes. Brief. Bioinform..

[89] Bailey J.A., Gu Z.P., Clark R.A., Reinert K., Samonte R.V., Schwartz S., Adams M.D., Myers E.W., Li P.W., Eichler E.E. (2002). Recent segmental duplications in the human genome. Science.

[90] Bailey J.A., Eichler E.E. (2006). Primate segmental duplications: crucibles of evolution, diversity and disease. Nat. Rev. Genet..

[91] Harrison P.M., Gerstein M. (2002). Studying genomes through the aeons: Protein families, pseudogenes and proteome evolution. J. Mol. Biol..

[92] Zhang Z.D., Frankish A., Hunt T., Harrow J., Gerstein M. (2010). Identification and analysis of unitary pseudogenes: Historic and contemporary gene losses in humans and other primates. Genome Biol..

[93] Zheng D., Gerstein M.B. (2006). A computational approach for identifying pseudogenes in the ENCODE regions. Genome Biol..

[94] Valdes C., Capobianco E. (2014). Methods to detect transcribed pseudogenes: RNA-Seq discovery allows learning through features. Methods Mol. Biol..

[95] Khurana E., Lam H.Y.K., Cheng C., Carriero N., Cayting P., Gerstein M.B. (2010). Segmental duplications in the human genome reveal details of pseudogene formation. Nucleic Acids Res..

[96] Dahia P.L.M., FitzGerald M.G., Zhang X., Marsh D.J., Zheng Z.M., Pietsch T., von Deimling A., Haluska F.G., Haber D.A., Eng C. (1998). A highly conserved processed PTEN pseudogene is located on chromosome band 9p21. Oncogene.

[97] Poliseno L., Salmena L., Zhang J., Carver B., Haveman W.J., Pandolfi P.P. (2010). A coding-independent function of gene and pseudogene mRNAs regulates tumour biology. Nature.

[98] Karreth F.A., Reschke M., Ruocco A., Ng C., Chapuy B., Leopold V., Sjoberg M., Keane T.M., Verma A., Ala U. (2015). The BRAF pseudogene functions as a competitive endogenous RNA and induces lymphoma in vivo. Cell.

[99] Karro J.E., Yan Y., Zheng D., Zhang Z., Carriero N., Cayting P., Harrrison P., Gerstein M. (2007). Pseudogene.org: A comprehensive database and comparison platform for pseudogene annotation. Nucleic Acids Res..

[100] Lam H.Y.K., Khurana E., Fang G., Cayting P., Carriero N., Cheung K.-H., Gerstein M.B. (2009). Pseudofam: The pseudogene families database. Nucleic Acids Res..

[101] Lee R.C., Feinbaum R.L., Ambros V. (1993). The C. elegans heterochronic gene lin-4 encodes small RNAs with antisense complementarity to lin-14. Cell.

[102] Morin R.D., O’Connor M.D., Griffith M., Kuchenbauer F., Delaney A., Prabhu A.L., Zhao Y.J., McDonald H., Zeng T., Hirst M. (2009). Application of massively parallel sequencing to microRNA profiling and discovery in human embryonic stem cells. Genome Res..

[103] Kozomara A., Griffiths-Jones S. (2014). mirBase: Annotating high confidence microRNAs using deep sequencing data. Nucleic Acids Res..

[104] Agarwal V., Bell G.W., Nam J.W., Bartel D.P. (2015). Predicting effective microRNA target sites in mammalian mRNAs. eLife.

[105] Berezikov E., Robine N., Samsonova A., Westholm J.O., Naqvi A., Hung J.H., Okamura K., Dai Q., Bortolamiol-Becet D., Martin R. (2011). Deep annotation of Drosophila melanogaster microRNAs yields insights into their processing, modification, and emergence. Genome Res..

[106] Rajagopalan R., Vaucheret H., Trejo J., Bartel D.P. (2006). A diverse and evolutionarily fluid set of micrornas in Arabidopsis thaliana. Genes Dev..

[107] Westholm J.O., Ladewig E., Okamura K., Robine N., Lai E.C. (2012). Common and distinct patterns of terminal modifications to mirtrons and canonical microRNAs. RNA.

[108] Larter C.Z., Yeh M.M. (2008). Animal models of NASH: Getting both pathology and metabolic context right. J. Gastroenterol. Hepatol..

[109] Bartel D.P. (2004). MicroRNAs: Genomics, biogenesis, mechanism, and function. Cell.

[110] van Rooij E., Sutherland L.B., Qi X., Richardson J.A., Hill J., Olson E.N. (2007). Control of stress-dependent cardiac growth and gene expression by a microRNA. Science.

[111] Xiao C., Calado D.P., Galler G., Thai T.H., Patterson H.C., Wang J., Rajewsky N., Bender T.P., Rajewsky K. (2007). MiR-150 controls B cell differentiation by targeting the transcription factor c-Myb. Cell.

[112] Guarnieri D.J., DiLeone R.J. (2008). MicroRNAs: A new class of gene regulators. Ann. Med..

[113] Wen Z., Zhang J., Tang P., Tu N., Wang K., Wu G. (2017). Overexpression of mir185 inhibits autophagy and apoptosis of dopaminergic neurons by regulating the ampk/mtor signaling pathway in parkinson’s disease. Mol. Med. Rep..

[114] Casanova-Salas I., Rubio-Briones J., Calatrava A., Mancarella C., Masia E., Casanova J., Fernandez-Serra A., Rubio L., Ramirez-Backhaus M., Arminan A. (2014). Identification of miR-187 and miR-182 as biomarkers of early diagnosis and prognosis in patients with prostate cancer treated with radical prostatectomy. J. Urol..

[115] Kumar B., Lupold S.E. (2016). MicroRNA expression and function in prostate cancer: A review of current knowledge and opportunities for discovery. Asian J. Androl..

[116] Calin G.A., Croce C.M. (2006). MicroRNAs and chromosomal abnormalities in cancer cells. Oncogene.

[117] Garcia-Silva M.R., Cabrera-Cabrera F., Guida M.C., Cayota A. (2012). Hints of tRNA-derived small RNAs role in RNA silencing mechanisms. Genes.

[118] Fu H., Feng J., Liu Q., Sun F., Tie Y., Zhu J., Xing R., Sun Z., Zheng X. (2009). Stress induces tRNA cleavage by angiogenin in mammalian cells. FEBS Lett..

[119] Harada F., Dahlberg J.E. (1975). Specific cleavage of tRNA by nuclease S1. Nucleic Acids Res..

[120] Nekrasov M.P., Ivshina M.P., Chernov K.G., Kovrigina E.A., Evdokimova V.M., Thomas A.A.M., Hershey J.W.B., Ovchinnikov L.P. (2003). The mRNA-binding protein YB-1 (p50) prevents association of the eukaryotic initiation factor eIF4G with mRNA and inhibits protein synthesis at the initiation stage. J. Biol. Chem..

[121] Bakowska-Zywicka K., Kasprzyk M., Twardowski T. (2016). tRNA-derived short RNAs bind to *Saccharomyces cerevisiae* ribosomes in a stress-dependent manner and inhibit protein synthesis in vitro. FEMS Yeast Res..

[122] Yamasaki S., Ivanov P., Hu G.F., Anderson P. (2009). Angiogenin cleaves tRNA and promotes stress-induced translational repression. J. Cell Biol..

[123] Guo Y., Bosompem A., Mohan S., Erdogan B., Ye F., Vickers K.C., Sheng Q., Zhao S., Li C.I., Su P.F. (2015). Transfer RNA detection by small RNA deep sequencing and disease association with myelodysplastic syndromes. BMC Genom..

[124] Abbott J.A., Francklyn C.S., Robey-Bond S.M. (2014). Transfer RNA and human disease. Front. Genet..

[125] Anderson P., Ivanov P. (2014). tRNA fragments in human health and disease. FEBS Lett..

[126] Fu Y., Lee I., Lee Y.S., Bao X. (2015). Small non-coding transfer RNA-derived RNA fragments (tRFs): Their biogenesis, function and implication in human diseases. Genom.Inform..

[127] Mishima E., Inoue C., Saigusa D., Inoue R., Ito K., Suzuki Y., Jinno D., Tsukui Y., Akamatsu Y., Araki M. (2014). Conformational change in transfer RNA is an early indicator of acute cellular damage. J. Am. Soc. Nephrol..

[128] Zheng G., Qin Y., Clark W.C., Dai Q., Yi C., He C., Lambowitz A.M., Pan T. (2015). Efficient and quantitative high-throughput tRNA sequencing. Nat. Methods.

[129] Seto A.G., Kingston R.E., Lau N.C. (2007). The coming of age for Piwi proteins. Mol. Cell.

[130] Ross R.J., Weiner M.M., Lin H. (2014). PIWI proteins and PIWI-interacting RNAs in the soma. Nature.

[131] Ng K.W., Anderson C., Marshall E.A., Minatel B.C., Enfield K.S., Saprunoff H.L., Lam W.L., Martinez V.D. (2016). Piwi-interacting RNAs in cancer: emerging functions and clinical utility. Mol. Cancer.

[132] Guo X., Zhang Z., Gerstein M.B., Zheng D. (2009). Small RNAs originated from pseudogenes: cis- or trans-acting?. PLoS Comput. Biol..

[133] Parrish N.F., Fujino K., Shiromoto Y., Iwasaki Y.W., Ha H., Xing J., Makino A., Kuramochi-Miyagawa S., Nakano T., Siomi H. (2015). piRNAs derived from ancient viral processed pseudogenes as transgenerational sequence-specific immune memory in mammals. RNA.

[134] Zhang A.T., Langley A.R., Christov C.P., Kheir E., Shafee T., Gardiner T.J., Krude T. (2011). Dynamic interaction of Y RNAs with chromatin and initiation proteins during human DNA replication. J. Cell Sci..

[135] Hizir Z., Bottini S., Grandjean V., Trabucchi M., Repetto E. (2017). RNY (YRNA)-derived small RNAs regulate cell death and inflammation in monocytes/macrophages. Cell Death Dis..

[136] Valadkhan S., Gunawardane L.S. (2013). Role of small nuclear RNAs in eukaryotic gene expression. Essays Biochem..

[137] Henry R.W., Mittal V., Ma B., Kobayashi R., Hernandez N. (1998). SNAP19 mediates the assembly of a functional core promoter complex (SNAP_c_) shared by RNA polymerases II and III. Genes Dev..

[138] Falaleeva M., Pages A., Matuszek Z., Hidmi S., Agranat-Tamir L., Korotkov K., Nevo Y., Eyras E., Sperling R., Stamm S. (2016). Dual function of C/D box small nucleolar RNAs in rRNA modification and alternative pre-mRNA splicing. Proc. Natl. Acad. Sci. USA.

[139] Bishop J.M., Levinson W.E., Sullivan D., Fanshier L., Quintrell N., Jackson J. (1970). The low molecular weight RNAs of Rous sarcoma virus. II. The 7 S RNA. Virology.

[140] Shan S.O., Walter P. (2005). Co-translational protein targeting by the signal recognition particle. FEBS Lett..

[141] Abell B.M., Pool M.R., Schlenker O., Sinning I., High S. (2004). Signal recognition particle mediates post-translational targeting in eukaryotes. EMBO J..

[142] Peterlin B.M., Brogie J.E., Price D.H. (2012). 7SK snRNA: A noncoding RNA that plays a major role in regulating eukaryotic transcription. Wiley Interdiscip. Rev. RNA.

[143] Capece V., Garcia Vizcaino J.C., Vidal R., Rahman R.U., Pena Centeno T., Shomroni O., Suberviola I., Fischer A., Bonn S. (2015). Oasis: Online analysis of small RNA deep sequencing data. Bioinformatics.

[144] Vitsios D.M., Enright A.J. (2015). Chimira: Analysis of small RNA sequencing data and microRNA modifications. Bioinformatics.

[145] Baras A.S., Mitchell C.J., Myers J.R., Gupta S., Weng L.C., Ashton J.M., Cornish T.C., Pandey A., Halushka M.K. (2015). miRge - a multiplexed method of processing small RNA-seq data to determine microRNA entropy. PLoS ONE.

[146] Guo Y., Strickland S.A., Mohan S., Li S., Bosompem A., Vickers K.C., Zhao S., Sheng Q., Kim A.S. (2017). MicroRNAs and tRNA-derived fragments predict the transformation of myelodysplastic syndromes to acute myeloid leukemia. Leuk. Lymphoma.

[147] Pantano L., Estivill X., Marti E. (2010). SeqBuster, a bioinformatic tool for the processing and analysis of small RNAs datasets, reveals ubiquitous miRNA modifications in human embryonic cells. Nucleic Acids Res..

[148] de Oliveira L.F., Christoff A.P., Margis R. (2013). isomiRID: A framework to identify microRNA isoforms. Bioinformatics.

[149] Zhang Y., Zang Q., Zhang H., Ban R., Yang Y., Iqbal F., Li A., Shi Q. (2016). DeAnniso: A tool for online detection and annotation of isomirs from small RNA sequencing data. Nucleic Acids Res..

[150] Selitsky S.R., Sethupathy P. (2015). tDRmapper: Challenges and solutions to mapping, naming, and quantifying tRNA-derived RNAs from human small RNA-sequencing data. BMC Bioinform..

